# Workplace Health Promotion in Washington State

**Published:** 2008-12-15

**Authors:** Jeffrey R. Harris, Patricia A. Lichiello, Peggy A. Hannon

**Affiliations:** Health Promotion Research Center; University of Washington, Seattle, Washington; University of Washington, Seattle, Washington

## Abstract

The workplace is a powerful setting to reach large numbers of at-risk adults with effective chronic disease prevention programs. Missed preventive care is a particular problem for workers with low income and no health insurance. The costs of chronic diseases among workers — including health care costs, productivity losses, and employee turnover — have prompted employers to seek health promotion interventions that are both effective and cost-effective. The workplace offers 4 avenues for delivering preventive interventions: health insurance, workplace policies, health promotion programs, and communications. For each of the avenues, the evidence base describes a number of preventive interventions that are applicable to the workplace. On the basis of the evidence and of our work in Washington State, we present a public health approach to preventing chronic diseases via the workplace. In addition to relying on the evidence, this approach makes a compelling business case for preventive interventions to employers.

## Introduction

The workplace offers an opportunity for chronic disease prevention. Chronic diseases largely result from health behaviors (1), and the workplace helps shape these behaviors through 4 important avenues: health insurance, workplace policies, health promotion programs, and communications. Health insurance affects workers' use of preventive care, such as tobacco use cessation programs and screening for colorectal cancer. Health insurance benefits offered by employers to workers and their dependents cover 158 million US residents and 59% of workers (2). Workplace policies can reduce harmful environmental exposures, such as exposure to secondhand smoke, and can increase access to physical activity facilities and healthy foods. Workplace programs, such as group physical activity programs and on-site influenza vaccinations, offer workers easy access to and social support for healthy activities. Workplace communications, such as e-mails, pamphlets, posters, and Web sites, can improve knowledge and shape beliefs, attitudes, and perceived norms about health behaviors and the health insurance benefits, policies, and programs aimed at improving them. Workplace communications can reach all workers, regardless of insurance coverage or program participation.

Employers increasingly recognize the financial effect of chronic diseases and the behaviors that cause them (3). Chronic diseases increase labor costs through many means, including health care costs, but also through productivity losses from missed work, decreased on-the-job effectiveness, and turnover when an employee becomes too ill to return to work (4). Since 2001, the cost to employers of providing health insurance has increased by 78% (2). Available data suggest that, for chronic diseases, the cost of productivity losses exceeds the cost of health care by as much as 4-fold (4).

To mitigate the effect of chronic diseases on employee productivity and decrease health care costs, employers are increasingly implementing workplace health promotion efforts (5). Much progress has been made in these efforts (6), but they can fall short in 2 ways. First, employers often choose interventions that are ineffective or unnecessary. Examples are the widespread use of health risk appraisals to change worker health behaviors and the use of hypertension screening in the workplace. Health risk appraisals alone do not change behaviors (6). Hypertension screening is so well-implemented in clinical care that very few need it provided elsewhere (7). (Author analyses of 1999 data from the Behavioral Risk Factor Surveillance System [BRFSS], the most recent available for hypertension screening, show that more than 90% of working adults aged 18 to 64 years, nationally and in Washington State, were screened for hypertension within the previous 2 years [8]). Second, employers often fail to choose effective interventions that offer the most value, measured in health outcomes and cost-effectiveness, for the preventive care dollar invested. For example, tobacco use cessation treatment and influenza vaccinations offer good return on investment but are fully implemented in less than a quarter of workplaces (5).

A public health approach to workplace health promotion can solve both of these problems (9). This approach is commonly used by the Centers for Disease Control and Prevention (CDC) and public health researchers and practitioners to frame efforts in a variety of public health issue areas. For the workplace, the approach involves 5 stages: 1) defining the problem, in this case, chronic diseases among workers; 2) elucidating risk factors, in this case, behaviors; 3) identifying effective interventions, 4) implementing suitable interventions, and 5) evaluating effectiveness.

For the past 6 years in Washington State, we have worked with employers, health departments, nonprofit organizations, and wellness program vendors to develop this 5-stage approach to preventing chronic diseases via the workplace and to make a compelling business case for its use. We summarize here information from both national and Washington-specific sources about each of the 5 stages and then offer conclusions.

## 1. Defining the problem

In Washington, among working-age adults aged 20 to 64 years, 4 chronic diseases — cancer, heart disease, diabetes, and chronic lower respiratory disease — account for more than 50% of deaths (10). Data are not available for chronic disease prevalence specifically among workers in Washington or nationwide. A large study of all active and retired workers at General Motors revealed that chronic diseases are also leading disabling conditions (11).

## 2. Elucidating risk factors

To prevent chronic diseases among working-age adults, we look to 3 authoritative sources that provide a short list of health behaviors of importance to chronic disease:

**CDC.** CDC placed 4 risk behaviors for chronic diseases at the top of the list of actual causes of death in 2000: tobacco use (435,000 deaths per year), physical inactivity, unhealthy eating, and overweight/obesity (the latter 3 together account for 365,000 deaths per year) (1).

**The United States Preventive Services Task Force (USPSTF).** USPSTF recommends that working-age adults of average risk receive screening and follow-up treatment for 7 chronic health problems: hypertension, lipid disorders, obesity, tobacco use, and breast, cervical, and colorectal cancers (12).

**The Advisory Committee on Immunization Practices (ACIP). **ACIP recommends annual influenza vaccination to those older than age 50 because of their age-related risk of chronic diseases and the risk of influenza-related exacerbation of these diseases (13).

To help make the business case for the employers' role in improving these health behaviors, the National Commission on Prevention Priorities has ranked preventive care services by health impact and cost-effectiveness (14). The Commission also analyzed delivery gaps and the potential for saving lives by filling these gaps (7). The rankings highlighted 3 types of preventive care as particularly cost-effective, and the gap analysis found that substantial reductions in death would result from their increased use: tobacco use screening and cessation programs (42,000 deaths averted annually), colorectal cancer screening (14,000), and influenza vaccination (12,000).

How common are these behaviors among workers? The BRFSS offers timely answers on a state-specific basis (Table 1, which includes the authors' analyses of BRFSS data) (8). Among workers in Washington, lifestyle risks are common: 76.3% do not eat enough fruits and vegetables, and 61.7% are overweight or obese. Little variation is seen between the insured and uninsured and among income groups for these lifestyle risks. Smoking, however, is affected by access to cessation treatment and is twice as common among the uninsured as among the insured (32.4% vs 14.8%) and nearly 3 times as common among those with annual household incomes less than $25,000 as among those earning more than $75,000 (28.8% vs 9.6%).

Similarly, Washington workers' use of recommended preventive care is too low (Table 1). Among workers aged 50 to 64 years, 61.6% have not had an influenza vaccination in the past year and 43.0% have missed colorectal cancer screening. The data reveal marked disparities by insurance status and income. Missed breast cancer screening is more than twice as common among the uninsured as among the insured (57.4% vs 22.1%) and among those in the bottom income group as among the top (45.4% vs 19.9%). Missed cervical cancer screening follows a similar pattern.

These findings from the BRFSS suggest that workers at all levels of income and access to care have similar needs for interventions to improve diet, physical activity, and weight. All workers could increase use of preventive care, although workers in low-income jobs and those with poor insurance coverage especially need interventions to improve use of key clinical preventive services.

## 3. Identifying effective interventions for the workplace

*The Guide to Community Preventive Services* (*Guide*) recommends 18 interventions that are either specifically conducted in the workplace or are readily adaptable to it. These 18 interventions are effective at promoting the 7 preventive behaviors for chronic disease discussed earlier (Table 2) (15). Eleven of the interventions are generalizable to multiple behaviors in our list, making them more broadly useful. The *Guide*'s insurance benefit interventions decrease financial barriers to preventive care by reducing out-of-pocket costs. They also build accountability through reminder systems for patients and providers and assessment and feedback systems for providers. Employers, particularly self-insured employers, can mandate these interventions by incorporating them into a health insurance benefit package. The *Guide*'s policy interventions make it easier to be physically active at work (through availability of facilities) and harder to smoke around coworkers (through restrictions and bans). The *Guide*'s programmatic interventions improve the social desirability of preventive behaviors (through group activities) and reduce barriers to care (through on-site cancer screening and telephone-based tobacco cessation counseling). The *Guide*'s communication interventions use virtually all types of media and can be tailored to the communication resources and channels available to different types of workplaces.

## 4. Implementing suitable interventions for the workplace

Washington, an average-sized state, has more than 104,000 employers with at least 2 workers (Table 3) (16,17). To implement effective workplace interventions, we need to understand the characteristics of these employers that affect how they can promote health. We have found that 2 characteristics — size (number of workers) and industry group — are associated with the health promotion resources available to employers and their workers.

Size is associated with the types of health promotion an employer delivers and the number of workers that such efforts reach. A 2001 national survey found that larger employers in the United States are more likely to cover preventive care and offer health promotion programs than are smaller employers (5). Larger employers can reach more workers not only because they individually employ large numbers of people but also because their size translates into greater purchasing power in the health insurance and health promotion marketplace. Larger employers also are more likely to have staff dedicated to human resources and to workplace health promotion. They also are more likely to use external advice to design health insurance benefits (18). Benefits consultants are the most common source of external advice for larger employers; insurance brokers are almost the exclusive source for smaller employers (18).

In Washington, the proportion of total workers is equal for 3 size-groups of employers: those with 2 to 49 workers, 50 to 499, and 500 or more (Table 3). Although most employers, regardless of size, offer health insurance to their full-time workers and dependents, larger employers are more likely to do so. Larger employers in the state also are much more likely to self-insure for the cost of their workers' health care, and doing so allows them to choose the design of the health insurance benefits they offer.

Another important characteristic of employers is industry (Table 4) (16,19). Government is the largest industry in Washington, employing nearly one-fifth of all workers. Retail trade and health care follow; each employs approximately 1 in 10 workers. The relationship between industry and wages also offers information for designing workplace health promotion interventions. Among industry groups in Washington, wages vary nearly 7-fold, with the highest in the information industry and the lowest in 2 groups: 1) accommodation and food services, and 2) agriculture, forestry, fishing, and hunting. Health insurance offerings increase with wages. At least 60% of employers in most industries offer health insurance to their full-time workers. In 6 of the 7 highest-paying industries, however, more than 80% do so. In only 2 groups — the 2 at the lowest end of the wage spectrum — do fewer than half offer insurance to their full-time workers.

Taken together, our findings can guide health promotion efforts. Government and other large employers, by virtue of their size, visibility, and strength in the marketplace, are primary targets for health promotion activities. But smaller employers employ most workers. Because of their limited resources, smaller employers are unlikely to offer extensive workplace health promotion services. Small employers are too numerous to be approached one by one, so intermediaries, such as chambers of commerce and insurance brokers, are channels to reach them. Employers in low-wage industries offer a specific opportunity to reach uninsured workers and their dependents by focusing on publicly available services, such as state-funded tobacco use cessation quitlines and federally and state-funded breast and cervical cancer screening programs.

## 5. Evaluating effectiveness

Workplace interventions should be carefully evaluated to determine effectiveness and identify areas for improvement. Evaluations of workplace interventions, however, have 3 common pitfalls. First, employee behavior is often measured via health risk appraisals that have low participation rates and overrepresentation of health-conscious workers (6,20). Second, participants are often compared with nonparticipants, resulting in strong selection bias and overestimation of intervention effect. Third, workplace evaluation efforts often focus either on 1) inputs such as program delivery that do not capture participation and behavior change or 2) disease outcomes, which are slow to change and may be affected by factors outside the intervention.

Researchers working with employers can use more rigorous evaluation designs to create and test new workplace interventions. Randomized experimental designs in large workplaces, wherein some workers will not receive the intervention, can present recruitment challenges. Working with smaller employers, however, researchers can randomize at the level of workplace rather than employee. Such cluster-randomized trials have the advantage of recruiting employers with truly independent workplace cultures and environments.

Practitioners working with employers can offer more effective workplace evaluation by continually tracking intervention delivery and employee participation and by measuring relevant behavior change. These data increase the probability of intervention success by catching any problems early enough to make midcourse corrections (21). Employers also will be more motivated to support effective interventions if they can see that the resources going to these interventions result in program participation and behavior change.

## Conclusions

The workplace offers an opportunity to reach large numbers of at-risk adults with effective chronic disease prevention programs. Chronic diseases are at the top of the list of employers' concerns and will only rise in importance as the baby boomers and the overall workforce age. Even workers with health insurance commonly have risk behaviors, and missed preventive care is a particular problem for workers with low incomes and no health insurance.

To address these behaviors, the evidence base provides a large number of effective interventions applicable to the workplace, but these interventions are underused. Barrier-reduction interventions, such as eliminating out-of-pocket costs for tobacco use cessation, should take priority in our efforts for 2 reasons. First, they have the broadest reach — to workers, dependents, and retirees — and can affect all workers, even those who do not participate in interventions. Second, financial barriers are most important for low-income workers with limited means.

Our description of the workplace world addresses a gap in practical information about working with employers. We must work with employers of all sizes and will need to tailor our approaches to their differing circumstances. Paradoxically, employers with the fewest resources offer an opportunity to reach uninsured low-income workers and link them with existing public health programs.

Two areas require further thought and action. First, although chronic diseases are important to employers, so are other health issues. A short list includes depression, musculoskeletal injuries, pregnancy, sleep disorders, stress, and substance abuse. Employers may ask us to address these issues at the same time as we address chronic diseases — and we need to be responsive to this request. Second, we have focused here on primary and secondary prevention of chronic diseases, but tertiary prevention is also needed, particularly for workers with diabetes, hypertension, and lipid disorders. Improved management of these diseases offers a public health opportunity for decreasing disease, death, health care costs, and productivity losses. Developing a public health approach for both of these issues will help employers use their health promotion dollars wisely and effectively.

## Acknowledgments

This article was sponsored by the University of Washington Health Promotion Research Center, one of CDC's Prevention Research Centers (HPRC cooperative agreement no. U48/DP000050, 03). Additional funding support came from CDC and the National Cancer Institute through the Cancer Prevention and Control Research Network, a network within CDC's Prevention Research Centers Program (Grant 1, U48, DP, 000050), and the CDC Office of Public Health Research through its Centers of Excellence in Health Marketing and Health Communication program (Grant 5, P01, CD000249, 02).

## Figures and Tables

**Table 1:** Prevalence of Risk Behaviors Among Employed Residents of Washington State by Insurance Status and Income, Washington State Behavioral Risk Factor Surveillance System, 2005 and 2006

Variable	% of Respondents^a^ (95% CI)							

	**Lifestyle Behaviors^b^**				**Preventive Care Behaviors^b^**			

	Inadequate Fruit and Vegetable Consumption	Inadequate Physical Activity	Overweight or Obese	Smoking	No Breast Cancer Screening	No Cervical Cancer Screening	No Colorectal Cancer Screening	No Influenza Vaccination
**Total**	76.3(75.3-77.3)	44.4(43.2-45.6)	61.7(60.4-63.0)	17.5(16.5-18.5)	24.8(23.2-26.5)	12.2(10.9-13.6)	43.0(41.1-44.9)	61.6(59.7-63.5)
**Insurance status**								
Uninsured	80.4(77.4-83.1)	51.0(47.2-54.8)	61.1(57.0-65.1)	32.4(28.7-36.3)	57.4(50.8-63.8)	27.7(22.8-33.3)	72.3(65.8-78.1)	82.3(76.5-86.9)
Insured	75.6(74.5-76.6)	43.3(42.0-44.6)	61.8(60.4-63.2)	14.8(13.9-15.8)	22.1(20.4-23.8)	9.8(8.6-11.2)	40.7(38.8-42.7)	60.0(58.0-62.0)
**Annual household income, $**								
<25,000	81.1(78.3-83.6)	51.8(48.2-50.4)	62.4(58.4-60.2)	28.8(25.3-32.6)	45.4(40.0-51.0)	17.9(14.4-22.0)	61.9(55.5-67.9)	73.3(67.4-78.5)
25,000- 49,999	78.3(76.4-80.0)	43.5(41.3-45.8)	64.4(61.9-66.8)	22.7(20.7-24.9)	27.7(24.4-31.2)	13.8(11.5-16.4)	48.3(44.5-52.1)	69.9(66.5-73.1)
50,000- 74,999	74.3(72.0-76.4)	43.9(41.3-46.4)	63.6(60.9-66.2)	16.5(14.4-18.9)	22.4(19.1-26.0)	10.8(8.0-14.4)	44.0(40.0-48.1)	60.7(56.8-64.6)
≥75,000	73.7(71.9-75.5)	40.8(38.7-42.9)	61.5(59.2-63.7)	9.6(8.3-11.1)	19.9(17.4-22.8)	6.0(4.6-7.8)	35.9(32.8-39.1)	55.4(52.1-58.7)

Abbreviation: CI, confidence interval.

a We calculated rates from Behavioral Risk Factor Surveillance System data for Washington State (8), 2005 (inadequate fruit and vegetable consumption and inadequate physical activity) and 2006 (all other behaviors). We restricted all analyses to employed respondents aged 18 to 64 years; total sample sizes for this subgroup were 11,724 for 2005 and 11,356 for 2006. The analyses took the complex survey design and weighted sampling probabilities of the data into account and were performed using SUDAAN statistical software version 9.01 (RTI International, Research Triangle Park, North Carolina).

b We defined outcome variables as follows: Inadequate fruit and vegetable consumption, consuming fewer than 5 servings of fruit and vegetables per day. Inadequate physical activity, not meeting guidelines for moderate physical activity (at least 5 sessions per week of at least 30 minutes each) or vigorous physical activity (at least 3 sessions per week of at least 20 minutes each). Overweight or obese, body mass index ≥25 kg/m^2^. Smoking, currently smokes. No breast cancer screening, women aged 40 to 64 years who have not had a mammogram in the past 2 years. No cervical cancer screening, women who have not had a Papanicolaou test in the past 3 years. No colorectal cancer screening, adults aged 50 to 64 years who have had neither a fecal occult blood test in the past year nor an endoscopy (flexible sigmoidoscopy or colonoscopy) in the past 10 years. No influenza vaccination, adults aged 50 to 64 years who have neither had a flu shot nor nasal flu spray vaccination in the past year.

**Table 2:** *Guide to Community Preventive Services* a Intervention Recommendations Relevant to the Workplace

Interventions by Type	Behavior Addressed						

	Breast Cancer Screening	Cervical Cancer Screening	Colorectal Cancer Screening^b^	Influenza Vaccination	Tobacco Use Cessation and Secondhand Smoke Exposure	Physical Activity	Weight Management
**Insurance Benefits**							
Client (enrollee) reminders^c^	X	X	X	X			
Client (enrollee) incentives, with reminders^c^	X						
Provider assessment and feedback^c^	X	X	X	X			
Provider reminders, with or without provider education^c^	X	X	X	X	X		
Reduce out-of-pocket expenditures^c^	X			X	X		
Multicomponent interventions to expand access in health care settings				X			
**Policies**							
Creation of, and/or enhanced access to, places for physical activity, informational outreach activities						X	
Smoking bans and restrictions					X		
**Programs**							
Multicomponent interventions, using education, enhanced access, and media^c^	X	X		X			X
Workplace screening (reduce structural barriers)^c^	X		X				
Physical activity interventions, individually adapted						X	
Physical activity interventions, with social support						X	
Tobacco quitlines (telephone support) combined with other interventions					X		
**Communications**							
Community-wide campaigns^c^						X	
Mass media combined with other interventions^c^					X		
One-on-one education^c^	X	X					
Small media^c^	X	X	X				
Use-the-stairs reminders (point-of-decision prompts)						X	

X indicates that the intervention has sufficient or strong evidence of effectiveness for the behaviors indicated.

a Source: Task Force on Community Preventive Services (15).

b The US Preventive Services Task Force recommends 3 methods for colorectal cancer screening: fecal occult blood test (FOBT), sigmoidoscopy alone or in combination with FOBT, and colonoscopy.

c The intervention is generalizable to other behaviors. "Small media" is defined as videos or printed communications that are distributed from health care systems or other community settings and convey educational or motivational information to promote the desired behavior.

**Table 3:** Employer Characteristics by Size, Washington State, 2005 and 2006^a^

No. of Employees	No. of Companies, (%)^b^	Proportion of All Employees^b^	% Offering Health Insurance to			Proportion of Companies That Self-Insure^c^

			Full-Time Employees^b^	Part-Time Employees^b^	Dependents of Full-Time Employees^b^	
2-9	70,631 (67.8)	12.0	59.9	11.2	44.5	13.5
10-24	19,585 (18.8)	11.8	72.4	15.6	61.8	
25-49	7,084 (6.8)	9.9	82.4	18.6	75.4	
50-99	3,438 (3.3)	9.7	91.3	21.4	87.3	
100-499	2,813 (2.7)	22.5	96.0	34.9	94.6	38.9
≥500	521 (0.5)	34.0	99.4	60.8	99.3	83.6
**Total**	104,072 (100)	100	66.4	14.2	53.6	33.2

a Data sources: Lockhart (16) and Agency for Healthcare Research and Quality (17).

b Limited to companies covered by the state's unemployment insurance law that have ≥2 employees, and are not government, except education and health care (16).

c Includes companies that have fewer than 100, 100-499, or 500 or more employees (17).

**Table 4:** Employer Characteristics by Industry Division, Washington State, 2006^a^

Industry	Average Annual Wage, $^b^	Proportion of All Employees, %^b^	% Offering Health Insurance to	

			Full-Time Employees, %^c^	Dependents of Full-Time Employees, %^c^
Accommodation and food services	15,469	7.8	25.5	16.6
Agriculture, forestry, fishing, and hunting	22,239	2.9	32.0	23.5
Other services, except public administration	23,009	3.9	68.4	54.8
Arts, entertainment, and recreation	27,139	1.6	62.2	49.8
Retail trade	28,174	11.1	59.6	47.1
Educational services	30,901	1.0	74.8	59.8
Administrative and waste services	34,533	5.1	61.8	47.3
Real estate and rental and leasing	34,948	1.7	69.6	50.7
Health care and social assistance	37,654	10.1	75.3	58.3
Construction	43,746	6.4	64.0	50.9
Transportation and warehousing	44,078	2.9	70.6	59.7
Government	44,745	17.7	NA	NA
Mining	54,924	0.1	73.7	62.7
Wholesale trade	56,572	4.3	85.4	76.2
Manufacturing	58,196	9.9	81.1	72.0
Professional and technical services	63,687	5.0	84.6	68.3
Finance and insurance	66,684	3.6	83.8	71.8
Utilities	70,404	0.2	69.6	58.2
Management of companies and enterprises	85,031	1.2	92.7	89.7
Information	91,081	3.4	82.8	75.7
**Total**	42,888	100.0	66.4	53.6

Abbreviations: NA, not available.

a Data sources: Lockhart (16) and Washington State Employment Security Department (19).

b Limited to companies covered by the state's unemployment insurance law (19).

c Limited to companies covered by the state's unemployment insurance law that have ≥2 employees and are not government, except education and health care (16).

## References

[1] Mokdad AH, Marks JS, Stroup DF, Gerberding JL (2004). Actual causes of death in the United States, 2000. JAMA.

[2] (2007). Employer health benefits 2007 annual survey.

[3] Galvin RS, Delbanco S (2006). Between a rock and a hard place: understanding the employer mind-set. Health Aff (Millwood).

[4] Loeppke R, Taitel M, Richling D, Parry T, Kessler R, Hymel P (2007). Health and productivity as a business strategy. J Occup Environ Med.

[5] Bondi MA, Harris JR, Atkins D, French ME, Umland B (2006). Employer coverage of clinical preventive services in the United States. Am J Health Promot.

[6] Goetzel RZ, Ozminkowski RJ (2008). The health and cost benefits of work site health-promotion programs. Annu Rev Public Health.

[7] (2007). National Commission on Prevention Priorities. Preventive care: a national profile on use, disparities, and health benefits.

[8] (2008). BRFSS: Behavioral Risk Factor Surveillance System, 1984-2008.

[9] Satcher D, Higgenbotham E (2008). The public health approach to the elimination of disparities in health. Am J Public Health.

[10] Washington State Department of Health, Center for Health Statistics. Mortality Table C3. Leading causes by age group and sex for residents, 2005.

[11] Friedman C, McKenna MT, Ahmed F, Krebs JG, Michaud C, Popova Y (2004). Assessing the burden of disease among an employed population: implications for employer-sponsored prevention programs. J Occup Environ Med.

[12] Department of Health and Human Services. US Preventive Services Task Force (USPSTF).

[13] Advisory Committee on Immunization Practices (2007). Prevention and control of influenza. MMWR Recomm Rep.

[14] Maciosek MV, Coffield AB, Edwards NM, Flottemesch TJ, Goodman MJ, Solberg LI (2006). Priorities among effective clinical preventive services: results of a systematic review and analysis. Am J Prev Med.

[15] Task Force on Community Preventive Services. The guide to community preventive services.

[16] Lockhart R (2007). Washington State employee benefits report (2006).

[17] (2005). Center for Financing, Access and Cost Trends. Medical expenditure panel survey, insurance component. (Table II.A.2.a).

[18] Marquis MS, Long SH (2000). Who helps employers design their health insurance benefits?. Health Aff (Millwood).

[19] Washington State Employment Security Department, Labor Market and Economic Analysis Branch. Data Table 8509: Covered Employment Classified by Industry, Annual Averages 2006 (REVISED).

[20] Lynch WD, Gilfillan LA, Jennett C, McGloin J (1993). Health risks and health insurance claims costs: results for health hazard appraisal responders and nonresponders. J Occup Med.

[21] Centers for Disease Control and Prevention (1999). Framework for program evaluation in public health. MMWR Recomm Rep.

