# Erratum, Vol. 6: Issue 1

**Published:** 2009-03-15

Because of an editing error, a footnote incorrectly appeared in the article "Workplace Health Promotion in Washington State." The footnote followed Table 4 in the article. Corrections were made to our Web site on January 28, 2009, and appear online at www.cdc.gov/pcd/issues/2009/jan/07_0276.htm. We regret any confusion or inconvenience this error may have caused.

